# PIEZO Force Sensors and the Heart

**DOI:** 10.1101/cshperspect.a041806

**Published:** 2025-07-28

**Authors:** Anna McGrane, Michael Murray, Fiona Bartoli, Marilena Giannoudi, Marcella Conning-Rowland, Leander Stewart, Eylem Levelt, Richard M. Cubbon, Erica Dall’Armellina, Kathryn J. Griffin, Kate M. Herum, Andrew J. Smith, David J. Beech

**Affiliations:** 1 Leeds Institute of Cardiovascular and Metabolic Medicine, School of Medicine, University of Leeds, Leeds LS2 9JT, United Kingdom; 2 Department of Cardiology, Leeds Teaching Hospitals NHS Trust, Leeds LS2 9JT, United Kingdom; 3 Baker Heart and Diabetes Institute, Melbourne, Victoria 3004, Australia; 4 Department of Histopathology, Leeds Teaching Hospitals NHS Trust, Leeds LS2 9JT, United Kingdom; 5 Research and Early Development, NovoNordisk A/S, Maaloev 2760, Denmark; 6 School of Biomedical Sciences, Faculty of Biological Sciences, University of Leeds, Leeds LS2 9JT, United Kingdom

## Abstract

The PIEZO1 and PIEZO2 membrane proteins form uniquely structured calcium permeable nonselective cation channels dedicated to mechanical force sensing in eukaryotic cells. In this review of the scientific literature, we address PIEZOs in the heart. PIEZOs enable the formation of the aortic valve, cardiac vasculature, and pericardial drainage. In the established heart, they enable baroreceptor pressure sensing and reflex regulation of the heart rate and influence the heart’s size and stiffness through roles in cardiac myocytes and cardiac fibroblasts. Therefore, mechanical force sensing by PIEZOs participates in normal cardiac development and function. There is also interest in PIEZOs in pathophysiology, when the structure and mechanical properties of the heart often change. Studies in rats and mice suggest that experimentally induced cardiac stress and injury cause PIEZO upregulation that is adverse. Similar changes may occur in human heart disease, creating potential for therapeutic benefit through PIEZO modulation. This is a productive, accelerating, and exciting new research topic with importance for our understanding of the heart and its diseases.

The heart regulates its own mechanical suitability by sensing the mechanical forces it generates and experiences, yet the molecular mechanisms of this sensing have been challenging to understand (Peyronnet et al. 2016; Saucerman et al. 2019; Sheetz 2021; Powers and McCulloch 2022; Pesce et al. 2023).^7^ All molecules of the heart may respond to force and thereby adapt, but certain molecules in this panoply became apparent as specialist force sensors. Ion channels have been candidate sensors of this type because they are fast electrical signal generators and the heart responds quickly to mechanical stimuli with electrical events (Peyronnet et al. 2016; Saucerman et al. 2019). Plurality of ion channels in mechanical–electrical coupling has been suggested and there are certainly many types of ion channels involved (Friedrich et al. 2012; Izu et al. 2020), but potential proof for ion channels as specialist sensors has arisen with the identification of the extraordinary force-sensing ion channel subunits PIEZO1 and PIEZO2 (Coste et al. 2010; Murthy et al. 2017). Their discovery has been seminal in the research journey to understanding force sensing in biology as a whole (Coste et al. 2010), and it is now advancing understanding of the heart and its disorders. PIEZO channels are exceptional in their structures and capabilities (Jiang et al. 2021b; Liu et al. 2025), presenting primary force sensors as opposed to force-modulated proteins or proteins that are downstream from the primary sensors (Coste et al. 2010; Nickolls et al. 2022). Publicity for the Nobel Prize recognizing the discovery of PIEZOs emphasized PIEZO2 in touch sensation, but the PIEZOs and especially PIEZO1 are widely expressed in many cell types (Jiang et al. 2021b) (PIEZO1 [www.gtexportal.org/home/gene/PIEZO1], PIEZO2 [www.gtex/portal.org/home/gene/PIEZO2]). They may be universal force sensors of all eukaryotic cells (Jiang et al. 2021b) and therefore potentially all cells of the cardiovascular system (Li et al. 2014; Beech and Kalli 2019; Coste and Delmas 2024) with diverse implications (Jiang et al. 2021b; Coste and Delmas 2024). In this narrative review, we focus on PIEZOs in the heart and its proximity.

PIEZOs are large membrane proteins that assemble as trimers of three PIEZO1s or three PIEZO2s to form homomeric PIEZO1 or PIEZO2 ion channels conducting Ca^2+^, Na^+^, K^+^, and other ions (Coste et al. 2010, 2015; Guo and MacKinnon 2017; Jiang et al. 2021b). They respond in milliseconds to force and thereby generate rapid electrical signals that quickly alter cell functions through, for example, changes in membrane potential (Coste et al. 2010; Jiang et al. 2021b) and Ca^2+^ influx-activated calpain protease (Li et al. 2014). The PIEZO channels are dedicated to the detection of force (Nickolls et al. 2022), which is achieved through their unusual ability to locally indent the membrane and project tentacles into it (Guo and MacKinnon 2017; Haselwandter et al. 2022). Once activated, the channels can adapt to continued force, switching off the ion conduction pathway through a process called inactivation (Coste et al. 2010). The speed and scale of this inactivation is regulated and crucial in how the PIEZOs serve diverse needs of different cell types and contexts (Li et al. 2014; Del Mármol et al. 2018; Beech and Kalli 2019; Shi et al. 2020). Inactivation is regulated through lipids such as ceramide (Shi et al. 2020) and proteins such as MyoD family inhibitor domain-containing protein MDFIC (Zhou et al. 2023) and the cell adhesion protein CADM1 (Koster et al. 2024).

The PIEZO1 and PIEZO2 channels differ in their mechanical characteristics (Jiang et al. 2021b) and responses to modulators such as lipids (Gabrielle et al. 2024), suggesting that the two different PIEZOs facilitate integration of different cells into different mechanical and lipid environments. PIEZO1 channels seem to be more flexible in their mechanical force sensing than PIEZO2 channels. At least in experimental situations, they respond to cell indentation, pressure that stretches the membrane, cell substrate displacement, fluid flow, and traction force (Jiang et al. 2021b). PIEZO2 channels respond best to cell indentation (Jiang et al. 2021b), with faster and briefer activity than PIEZO1 channels (Coste et al. 2010).

The classes of cell in the heart are the atrial and ventricular cardiac myocytes, pacemaker and conduction cells, fibroblasts and myofibroblasts, valve, vascular and lymphatic endothelial cells, valve interstitial cells and osteoblasts, pericytes, vascular smooth muscle cells, tissue-resident macrophages, nerve cell components such as nerve terminals, blood cells including red blood cells and immune cells, epicardial adipocytes, and stem or stem-like cells. In these cell classes, there is heterogeneity and plasticity (Litviňuková et al. 2020; Tucker et al. 2020; Rood et al. 2025). Because PIEZOs are widely expressed, many cells of the heart potentially use PIEZOs for their biological functions, perhaps with the rare exception of the cardiac tissue-resident macrophages (Simon-Chica et al. 2024). Despite the broad expression of PIEZOs, there are variations across the different cell types in the abundances of PIEZO1 and PIEZO2, the PIEZO1 and PIEZO2 channel properties, and the contributions of PIEZOs relative to other force sensing or force-sensitive mechanisms.

In physiology, most cells experience mechanical forces such as those from tissue structure and viscoelasticity, cell–cell interaction, cell migration and infiltration, extracellular and intracellular matrix stiffness, blood or lymph flow through tubes and chambers, movement of body parts, adaptations driven by changing external demands, gravity and physical injury. In the heart, additional forces arise (Herum et al. 2017a) from the rhythmic contractions and pumping of blood, the torsional rotation of the ventricles during filling and ejection, the heart chamber pressures and fluid dynamics, the opening and closing of the heart valves, and the pressure in the pericardium. In aging and disease, further changes in the mechanical forces occur, due to, for example, increased tissue stiffness, pressure overload, compensated and decompensated hypertrophy, calcification, atherosclerosis, coronary artery spasm, ischemia, cell death, infarction, and fibrosis. It is a major challenge for researchers to address PIEZO contributions in such varied situations. These channels have relatively large ionic conductance that may greatly alter membrane ionic permeability, membrane potential, intracellular Ca^2+^ homeostasis, and action potential characteristics, so cells may need to tightly control their PIEZOs to avoid unwanted effects such as excess depolarization.

The research on this topic is expanding quickly. Here, we summarize current knowledge and discuss interpretations of the available data and ideas for future research. We begin with PIEZOs in the heart’s physiology and progress to the pathophysiological relevance and potential for therapeutic applications.

## Physiology

### Cardiac Valve Formation

*Piezo1* gene disruption in zebrafish was found to disturb outflow tract valve morphogenesis, indicating a requirement for PIEZO1 in valve formation (Duchemin et al. 2019). PIEZO1 was suggested to regulate valve formation by mediating a fluid shear stress effect on the endothelial cell layer via KLF2 and NOTCH1 proteins, and a stretch effect on the smooth muscle cell layer via YAP1. *Piezo2* gene disruption similarly disturbed valve formation, albeit less effectively, and the combined disruption of *Piezo1* and *Piezo2* almost prevented valve formation (Duchemin et al. 2019). In an independent zebrafish study when PIEZO1 was depleted rather than deleted, there was also disturbed outflow tract and valve formation (Faucherre et al. 2020).

Downstream from fluid flow–activated PIEZO1 channels in endothelial cells is NOTCH1 (Caolo et al. 2020), which is also required for aortic valve development (Garg et al. 2005). Consistent with PIEZO1 and NOTCH1 cooperating in valve formation, *PIEZO1* and *NOTCH1* gene variants associate with bicuspid aortic valve disease (Garg et al. 2005; Faucherre et al. 2020). Therefore, flow sensing by PIEZO1 and its downstream signaling to NOTCH1 seem to be conserved in valve formation from fish to humans. Consistent with the involvement of PIEZO1 in human valves, unidirectional fluid flow caused upregulation of *PIEZO1* messenger RNA (mRNA) and elevated PIEZO1-dependent intracellular Ca^2+^ increases in induced pluripotent stem cell–derived valve endothelial cells (Xie et al. 2024). In these endothelial cells, fluid flow–induced branching structures and nitric oxide production were both PIEZO1 dependent, and fluid flow contributed to the adhesion of these cells to decellularized pig heart valves in a PIEZO1-dependent manner (Xie et al. 2024). PIEZO1’s ability to drive the formation of nitric oxide (Li et al. 2014; Wang et al. 2016b; Lichtenstein et al. 2024), a critical signaling mediator of fluid flow responses (Lundberg and Weitzberg 2022), therefore, seems to be an additional factor in aortic valve formation (Lee et al. 2000).

### Coronary Vascular Development

A genetic engineering method to track transcription from the *Piezo2* gene in mice suggested PIEZO2 expression in cells of the sinus venosus and coronary plexus at embryonic days 11.5–13.5 (Pampols-Perez et al. 2025). From embryonic day 15.5 to postnatal day 2, expression was mainly in the coronary endothelial clusters of capillaries, with little expression in other cell types of the heart up to early adult stage (Pampols-Perez et al. 2025). Consistent with this expression profile, PIEZO2 channel-like fast on and off mechanically activated currents were observed in endothelial cells at embryonic days 13.5–18.5 (Pampols-Perez et al. 2025). In *Piezo2* disrupted hearts, the branching of the left coronary artery into the circumflex artery and the left anterior descending artery did not follow the normal pattern, and coronary vessels were more constricted (Pampols-Perez et al. 2025). Therefore, PIEZO2 has a role in coronary vascular development and function.

In contrast to *Piezo2* expression, *Piezo1* expression was observed at all developmental and adult stages (Pampols-Perez et al. 2025). Three kinetically distinct mechanically activated currents were observed in embryonic day 13.5–18.5 endothelial cells, suggesting that PIEZO2 channels occur alongside other mechanically activated channels such as PIEZO1 channels (Pampols-Perez et al. 2025). The specific relevance of these channels to the heart remains to be clarified, as yolk sac endothelial cells were used in these studies and PIEZO1 channels have widespread importance in endothelial cells of other, noncardiac vasculatures (Li et al. 2014; Wang et al. 2016b; Albarrán-Juárez et al. 2018; Lichtenstein et al. 2024; Lim et al. 2024). Endothelial-specific deletion of PIEZO1 at adult stage in mice reduced the density of capillaries in skeletal muscle but not cardiac muscle (Bartoli et al. 2022a). Therefore, in adult physiology, myocardial vasculature may not need PIEZO1 or PIEZO2.

### Pericardial Fluid Drainage

Although global *Piezo1* gene disruption in mice is lethal, embryos at midgestation are small and superficially normal (Li et al. 2014; Ranade et al. 2014a). These embryos show pericardial effusion (Ranade et al. 2014a), suggesting importance of PIEZO1 in the physiological control of fluid volume around the heart. Consistent with this observation, PIEZO1 depletion in zebrafish caused pericardial edema (Faucherre et al. 2020). Moreover, adolescent onset of pericardial effusion occurred in humans carrying a single disruptive *PIEZO1* gene variant (Ludlow et al. 2023). These data suggest a conserved physiological role of PIEZO1 in pericardial fluid homeostasis, acting to prevent excess fluid accumulation that may otherwise reduce diastolic filling and cardiac output. Homozygosity of the disruptive *PIEZO1* variant was associated with cardiac tamponade that required pericardiocentesis (Ludlow et al. 2023). The basis of this pericardial effect is not defined, but the effusion occurred in the context of a generalized lymphatic dysplasia, pointing to a lymphatic origin of the pathology (Ludlow et al. 2023).

### Baroreceptor Regulation of Heart Rate

Cells of the nodose–petrosal–jugular ganglia in mice express *Piezo1* mRNA or *Piezo2* mRNA exclusively, or simultaneously in 15% of cells (Zeng et al. 2018). Similarly, retrograde labeling experiments in carotid sensory neurons suggested that cells specifically forming baroreceptors express *Piezo1* or *Piezo2* mRNA (Zeng et al. 2018). Moreover, *Piezo1* and *Piezo2* gene disruption (i.e., PIEZO1 and PIEZO2 knockout) in nodose and petrosal ganglion neurons of mice prevented the normal baroreceptor reflex of decreased heart rate in response to increased systolic blood pressure, while *Piezo1* or *Piezo2* disruption alone had no effect (Zeng et al. 2018). Double knockout also prevented the aortic depressor nerve activity that normally occurs with elevated blood pressure and the heart rate increase caused by blood pressure lowering (Zeng et al. 2018). It also increased blood pressure variability (Zeng et al. 2018), as expected with impaired baroreceptor function. Direct stimulation of PIEZO2-positive baroreceptor afferents, achieved through engineered optical control, lowered blood pressure (Zeng et al. 2018). Therefore, although more research on this topic would be worthwhile (Stocker et al. 2019), PIEZOs seem to work together to enable physiological pressure sensing in the aortic arch, and thus the coupling of heart rate to blood pressure. Consistent with these ideas, other studies showed that activation of PIEZO2-positive nodose–petrosal–jugular neurons decreased heart rate in mice and that ablation of the PIEZO2 neurons eliminated the baroreceptor reflex (Min et al. 2019). PIEZO2-positive nerve terminals form claw-like structures extending along the outer edge of the smooth muscle layer of the aortic arch, neatly positioning PIEZO2 to detect increases in arterial diameter with each pressure pulse (Min et al. 2019).

### Suppression of Cardiomyopathy

In mice, genetic insertion of an artificial peptide tag in native PIEZO1 enabled the detection of PIEZO1 in T-tubules of ventricular cardiac myocytes (Jiang et al. 2021a). Although cardiac myocyte–specific deletion of PIEZO1 in 8-week-old mice had no effect (Jiang et al. 2021a; Xu et al. 2025), in 18-week-old mice the hearts were larger with dilated left ventricles and inter-muscular fibrosis, and there was decreased ejection fraction (Jiang et al. 2021a). Other adverse cardiac effects, including premature cardiac aging, have been observed in cardiac myocyte PIEZO1-deleted mice (Chen et al. 2025b; Yu et al. 2025). Therefore, physiological cardiac myocyte PIEZO1 seems to be protective against cardiomyopathy. An interaction of PIEZO1 has been suggested with SERCA, which is a key cardiac protein localized to the sarco/endoplasmic reticulum membrane of cardiac myocytes (Zhang et al. 2017). It is not entirely clear which membrane is home to the cardiac myocyte PIEZO1, but if the channels are in the sarcolemmal (plasma) membrane as expected, PIEZO1 may signal across to the endoplasmic reticular membrane.

Global homozygous *Piezo2* gene disruption is lethal in mice at the perinatal stage (Ranade et al. 2014b), which is a later stage than the mid-embryonic lethality of global *Piezo1* gene disruption (Li et al. 2014; Ranade et al. 2014a). This delay enabled the characterization of hearts of *Piezo2* knockout mice at birth, which revealed cardiac hypertrophy relative to body weight and increased left ventricular and interventricular septal thickness without outflow tract defects (Pampols-Perez et al. 2025). In adult mouse and rat heart, PIEZO2 was detected in some cardiac myocytes, but echocardiographic assessment after cardiac myocyte–specific *Piezo2* disruption revealed no differences in heart dimensions or functions in mice that were 8–10 weeks old (Kloth et al. 2022). Therefore, although PIEZO2 may also protect against myopathy, it might be because of roles it has in other cell types of the heart.

### Extracellular Matrix Regulation

The extracellular matrix that is essential for the heart’s structure and stiffness is secreted and regulated by cardiac fibroblasts, which are abundant in the heart, highly mechanically sensitive and interspersed between the parenchymal cells (Herum et al. 2017a). The matrix surrounds and interconnects myocardial cells, providing a scaf-fold for myocytes and nonmyocytes, distributing mechanical forces, transmitting mechanical signals to individual cells, and regulating fluid movement through the interstitial compartment.

In mouse ventricular fibroblasts and human atrial appendage fibroblasts, *Piezo1* mRNA was found to be ∼10 times more abundant than in cardiac myocytes, suggesting the importance of PIEZO1 in the cardiac fibroblast cell population (Blythe et al. 2019). In support of this idea, patch-clamp recordings from cardiac fibroblasts revealed PIEZO1-dependent mechanically activated channel currents with a pressure of ∼60 mmHg causing 50% activation (Blythe et al. 2019). The currents showed little or no inactivation under continued pressure, suggesting an adaptive modification of the channels in cardiac fibroblasts for sustained activity. MDFIC protein was identified as a binding partner of PIEZO1 partly through affinity-capture mass spectrometry studies of cardiac fibroblasts (Zhou et al. 2023). Although not studied specifically in cardiac fibroblasts, genetically engineered embryonic fibroblasts with carboxy-terminally truncated MDFIC showed small PIEZO1 channel currents that inactivated rapidly compared with those of wild-type fibroblasts expressing wild-type full-length MDFIC (Zhou et al. 2023). Depletion of wild-type MDFIC similarly reduced PIEZO1 channel currents and accelerated the rate of inactivation (Zhou et al. 2023). Therefore, MDFIC’s integration with PIEZO1 might be what enables sustained PIEZO1 activity in cardiac fibroblasts.

*Piezo1* mRNA was also readily detected in rat ventricular fibroblasts. Depletion of PIEZO1 protein in these cells increased the basal expression of *Ctgf* (Ploeg et al. 2021). Under cell stretch conditions, it also increased the expression of *Tnc* and *Acta2* and prevented cell stretch–evoked increases in the expression of *Nppb* and *Tgfb1* (Ploeg et al. 2021). *Ctgf* encodes CCN2, which is a connective tissue growth factor that regulates fibrogenesis (Daniels et al. 2009), while *Tnc* encodes tenascin C, which is an extracellular matrix molecule with roles in cardiac myocyte differentiation and angiogenesis (Imanaka-Yoshida 2012). *Acta2* encodes α-smooth muscle actin, which is an identifier protein of myofibroblasts in injury responses and scarring (Tarbit et al. 2019). *Nppb* encodes brain natriuretic peptide (BNP), which is secreted in response to myocardial wall stress (Tsuruda et al. 2002), while *Tgfb1* encodes transforming growth factor β1 (TGFB1), which is a stimulator of fibroblast differentiation to myofibroblasts (Tarbit et al. 2019). These are just a few of the many regulators of cardiac fibroblasts and extracellular matrix, but the effects on them suggest roles of cardiac fibroblast PIEZO1 in integrating cardiac structure with tissue stretch and stiffness (Herum et al. 2017b; Braidotti et al. 2024).

Cyclic stretch and TGFB1 stimulated PIEZO2 expression in cultured murine neonatal cardiac fibroblasts, and depletion of PIEZO2 in these fibroblasts reduced their migration, proliferation, and autophagy (Ding et al. 2024). PIEZO1-specific activation by a chemical agonist enhanced these effects (Ding et al. 2024), suggesting a potential role for PIEZO1 in enabling a mechanical upregulation of PIEZO2.

## Pathophysiology

### Baroreceptor Downregulation in Hypertension

Reduced baroreceptor sensitivity is a feature of hypertension and other cardiovascular diseases such as heart failure (Salah et al. 2025). The abundance of *Piezo1* and *Piezo2* mRNAs in nodose ganglia was found to be reduced in rats with spontaneous hypertension or hypertension caused by a nitric oxide synthase inhibitor or angiotensin II (Huo et al. 2021). PIEZO2 was more abundant and more obviously reduced in the hypertensive animals than PIEZO1. Consistent with these data, rapidly inactivating PIEZO2 channel-like currents of aortic baroreceptor nodose ganglion neurons were reduced in hypertension (Huo et al. 2021). Moreover, the normal reflex reduction in heart rate caused by a vasoconstrictor agonist was blunted by the depletion of PIEZO2 but not PIEZO1 (Huo et al. 2021). Downregulation of PIEZO2 was mediated by the interaction of PIEZO2 with N4BP2 (Huo et al. 2021), which targets proteins for ubiquitination and endocytosis (Pohl et al. 2021). In rats on high fat diet with fructose in the drinking water, there was hypertension associated with decreased *Piezo1* mRNA and increased *Piezo2* mRNA in the aortic arch (Cui et al. 2024).

### Cardiac Hypertrophy due to Aortic Narrowing

Aortic stenosis across its severities associates with adverse cardiac outcomes that may include heart failure (Mihatov and Pibarot 2024). In mice, physiological PIEZO1 protein expression was found to be relatively low in the heart and very low in isolated cardiac myocytes (Yu et al. 2022). However, after transverse aortic constriction imposed surgically for an aortic stenosis-like effect, there was pressure overload and left ventricular hypertrophy without ventricular de-compensation or heart failure. There was a six-fold increase in *Piezo1* mRNA in isolated cardiac myocytes (Yu et al. 2022). Fourteen days after the overload, there was an ∼1.5-fold increase in PIEZO1 protein abundance in the left ventricle and cardiac myocytes isolated from the ventricle (Yu et al. 2022). To test the role of this PIEZO1, cardiac myocyte–specific PIEZO1 deletion was induced at the adult stage. While there were no effects on baseline cardiac parameters, the increases in left ventricular mass and wall thickness induced by aortic constriction were abolished (Yu et al. 2022). There was failure of hypertrophy signaling mediated by Ca^2+^-CAMK2A, HDAC4, and MEF2A, but calcineurin signaling, not normally involved, was induced (Yu et al. 2022). Fibrosis was also inhibited, suggesting dependence of the fibrosis on cardiac myocyte PIEZO1 (Yu et al. 2022). In a separate study using abdominal aortic constriction in rats, there was also increased PIEZO1 expression in the heart, and no change in PIEZO2 (Li et al. 2024). A reduced localization of PIEZO1 with the CAV3 caveolin protein may contribute to increases in PIEZO1 channel activity (Li et al. 2024).

In mice stressed by transverse aortic constriction, β-adrenergic receptor stimulation, or angiotensin II receptor stimulation, there was upregulation of *Piezo2* mRNA and PIEZO2 protein in the left ventricle that correlated with the upregulation of the collagen COL1A1, periostin, and other indicators of fibrosis (Ding et al. 2024). Single-cell RNA sequencing revealed *Piezo2* mRNA particularly in a cluster of late-differentiating cardiac fibroblasts (Ding et al. 2024). The increased *Piezo2* mRNA occurred transcriptionally via *Piezo2* exon methylation (Jiang et al. 2021c) mediated by the *N*^6^-methyl-adenosine (m^6^A) reader protein YTHDF1 (Ding et al. 2024). Adeno-associated virus–mediated depletion of PIEZO2 or YTHDF1 in β-adrenergic receptor–stimulated mice increased left ventricular ejection fraction while decreasing interventricular septal thickness and collagen deposition (Ding et al. 2024). Cardiac myocyte–specific deletion of PIEZO2 in mice had a mild protective effect against increased ventricular wall thickness caused by angiotensin II (Kloth et al. 2022). Therefore, PIEZO2 may also contribute to adverse cardiac adaptation caused by aortic narrowing or neurohormonal excess.

### Cardiac Ischemia, Infarction, and Reduced Ejection Fraction

In heart failure with reduced or preserved ejection fraction, cardiac ischemia is often an underlying determinant (Elgendy et al. 2019). Ca^2+^ homeostasis is expected to be altered in ischemia because of the importance of ATPases in cellular ionic control and intracellular Ca^2+^ regulation specifically (Balaban 2002). Ca^2+^-activated calpains are important in ischemia (Yu et al. 2022) and downstream from PIEZO1-mediated Ca^2+^ entry (Li et al. 2014; Zhang et al. 2021; Su et al. 2023; Xu et al. 2025).

The effects of myocardial ischemia, ischemia-reperfusion, and infarction on PIEZOs and their function in the heart have been studied in mice, rats, and pigs through experiments in which there was ligation of the left anterior descending coronary artery (LAD). Increased *Piezo1* mRNA and PIEZO1 proteins were detected in rat hearts 4 or 8 weeks after ligation, and at 8 weeks were concomitant with a left ventricular ejection fraction below 50%, indicating potential relevance to heart failure (Liang et al. 2017). In a similar study of rats, there was increased PIEZO1 in the heart 1 and 4 weeks after ligation (Niu et al. 2022). In studies of LAD-ligated mice, there was increased *Piezo1* mRNA and PIEZO1 protein in the heart (Zhang et al. 2021; Su et al. 2023), including in ischemia-reperfusion (Wang et al. 2024; Xu et al. 2025). PIEZO1 was also found to be upregulated in infarcted compared with noninfarcted heart tissue (Xu et al. 2025). PIEZO1 mRNA and protein were upregulated 8 weeks after ligation in pigs when there was reduced left ventricular ejection fraction (Lu et al. 2025).

Depletion of PIEZO1 by injecting lentivirus expressing short hairpin RNA into infarcted myocardium partially rescued the reduced left ventricular injection fraction caused by LAD ligation in rats (Lu et al. 2025). Further studies suggested that cardiac myocyte–specific PIEZO1 deletion protects against the reduced ejection fraction and interventricular septal thickening seen in the LAD-ligated mice, but not the increased left ventricular mass or infarct size, or the increased extracellular matrix deposition or scarring (Su et al. 2023). The PIEZO1 deletion also protected against a ventricular tachycardia triggered by electrical stimulation and improved the survival of the mice (Su et al. 2023). In another study of mice subjected to LAD ligation, cardiac myocyte–specific deletion of PIEZO1 protected against increased left ventricular mass, cardiac myocyte area, left ventricular internal diameter in diastole or systole, fibrosis, and the decreased ejection fraction and fractional shortening of the left ventricle (Zhang et al. 2021). In a study in which reperfusion was enabled, cardiac myocyte–specific PIEZO1 deletion reduced the infarct size, the ventricular dilation, and hypertrophy and improved the survival rate of the animals (Xu et al. 2025). Therefore, with LAD ligation in mice, PIEZO1 in cardiac myocytes accounts for key adverse effects (Su et al. 2023). Conversely, cardiac myocyte–specific overexpression of PIEZO1 in nonligated mice reduced the ejection fraction and caused arrhythmia, which suggests that excess cardiac myocyte PIEZO1 alone can drive adverse cardiac events (Jiang et al. 2021a).

In LAD-ligated rats, the increased PIEZO1 expression correlated with increased myocardial fibrotic area, stiffness, and expression of myofibroblast indicators such as *Acta2* mRNA (Niu et al. 2022). In studies of neonatal rat cardiac fibroblasts in cell culture, PIEZO1 and cytosolic Ca^2+^ concentration increased with substrate stiffness, and PIEZO1 depletion reduced myofibroblast indicators (Niu et al. 2022), consistent with the idea that excess PIEZO1 activation in fibroblasts promotes fibrosis. Synergy between PIEZO1 and the ITGB1 integrin (Wang et al. 2024) was instrumental in the coupling of these fibroblasts to substrate stiffness (Niu et al. 2022), suggesting an interplay of PIEZO1 with the integrin mediators of mechanical regulation.

Deletion of endothelial PIEZO1 reduced plaque formation in the brachiocephalic (innominate) artery, aortic root, carotid artery, and thoracic aorta in mouse models of atherosclerosis (Albarrán-Juárez et al. 2018). Therefore, PIEZO1 may be a factor promoting coronary artery atheroma, which is the major cause of cardiac ischemia. PIEZO1 also has roles in white blood cells that contribute to plaque formation and stability, as reviewed elsewhere (Yuan et al. 2023).

*Piezo2* expression was found not to change in the left ventricle of mice subjected to LAD ligation (Zhang et al. 2021). However, in rats, LAD ligation increased *Piezo2* expression, which appeared from almost nothing and was prevented by a mechanically protective artificial patch (Zhu et al. 2022). *Piezo1* expression was, in contrast, greater, and its increased expression was also prevented by the patch (Zhu et al. 2022; Lu et al. 2025).

### Interleukin 6 (IL-6) in Cardiac Inflammation

Cardiac inflammation is a component of the underlying pathophysiological processes in heart diseases, including heart failure with reduced or preserved ejection fraction (Alcaide et al. 2024). Specifically, IL-6, a proinflammatory cytokine and mediator of cardiovascular risk (Mehta et al. 2024), is associated with myocardial infarction and heart failure (Katkenov et al. 2024). PIEZO1 channels have emerged as key regulators of IL-6 in the heart.

In cultured cardiac fibroblasts, depletion of PIEZO1 reduced IL-6 mRNA and secretion when the cells were grown on a soft but not hard substrate (Blythe et al. 2019). The soft substrate is more likely to reflect the environment of heart tissue, so the results suggest that PIEZO1 channels positively regulate IL-6. PIEZO1 overexpression, designed to mimic PIEZO1 upregulation observed in patients with atrial fibrillation, increased the stiffness of cultured cardiac fibroblasts and the stiffness of nearby fibroblasts that were not overexpressing PIEZO1 (Emig et al. 2021). The overexpressing cells secreted more IL-6, and the increased stiffness of the overexpressing and non-overexpressing cells was prevented by an IL-6 neutralizing antibody, suggesting that IL-6 signals between the cells to regulate fibroblast stiffness (Emig et al. 2021). In mice, increased IL-6 mRNA and other inflammatory mediators in cardiac ischemia and reperfusion were reduced by cardiac myocyte–specific PIEZO1 deletion, suggesting a role for cardiac myocyte PIEZO1 in regulating IL-6 (Xu et al. 2025).

In rats up to 6 weeks after LAD ligation, increases in *Piezo1* mRNA and PIEZO1 protein were found in the thoracic dorsal root ganglia (TDRG) that are a source of sensory innervation for the heart (Sun et al. 2024). TDRG-specific PIEZO1 depletion reduced the severity of the ventricular remodeling seen at 4 weeks after LAD ligation and decreased IL-6 in the TDRG and heart (Sun et al. 2024). Axonal transport of IL-6 to the heart from the TDRG was suggested to have an adverse influence on the heart after myocardial infarction (Sun et al. 2024). These data suggest that PIEZO1 in neurons is a factor in the adverse response of the heart to ischemia, acting via IL-6 (Sun et al. 2024, 2025; Chen et al. 2025a). Moreover, excess shear stress acting via PIEZO1 increased monocyte adherence to the aortic valve and caused inflammation (Baratchi et al. 2020). Transcatheter aortic valve replacement reduced this effect and the expression of IL-6 (Baratchi et al. 2020). Therefore, there are multiple cellular sources and roles of PIEZO1-regulated IL-6.

## Relevance To Patients

Although pathophysiological relevance of PIEZOs has mostly been suggested from animal studies in which disease-like features were artificially induced, there are indications of the importance of PIEZOs in naturally occurring human heart disease. In addition to *PIEZO1* gene variant associations with cardiac valve abnormality (Faucherre et al. 2020) and pericardial effusion (Ludlow et al. 2023), recapitulation of a human *PIEZO1* gain-of-function variant in mice caused mild cardiac hypertrophy and fibrosis (Bartoli et al. 2022b), and there is a case report that associated a potential gain-of-function *PIEZO1* variant with cardiomyopathy (Cui et al. 2021). In aortic valves of patients with calcific aortic valve disease, there was upregulated *PIEZO1* mRNA and PIEZO1 protein with no change in *PIEZO2* mRNA (Zhong et al. 2023). PIEZO1 was elevated in all layers of the valve, with the highest amount in the endothelium of the aortic side, colocalized with the endothelial marker platelet and endothelial cell adhesion molecule 1 (PECAM1) and the osteogenic marker Runt-related transcription factor 2 (RUNX2) (Zhong et al. 2023). Recapitulation of a human *PIEZO2* gain-of-function variant in mice increased heart weight relative to body weight and reduced heart length (Pampols-Perez et al. 2025). Comparison of RNA sequencing data for 29 nonfailing and 31 ischemic cardiomyopathy hearts suggested increased *PIEZO1* and *PIEZO2* mRNA in myopathy (Zhu et al. 2022; Lu et al. 2025). A comparison of five nonmyopathy human heart samples with 35 diverse human hypertrophic obstructive cardiomyopathy heart samples also suggested increased *PIEZO1* mRNA in myopathy (Jiang et al. 2021a). Upregulated *PIEZO1* mRNA and PIEZO1 proteins were also suggested in a separate study of human dilated cardio-myopathy heart samples, with no change in *PIEZO2* mRNA (Su et al. 2023). PIEZO1 channel currents in cardiac fibroblasts from patients (Blythe et al. 2019; Jakob et al. 2021) were found to be larger when the patients had atrial fibrillation (Jakob et al. 2021).

### Pharmacology

#### PIEZO Inhibition

The spider toxin Grammostola Mechanotoxin #4 inhibits PIEZO1 and PIEZO2 channels (Kinsella et al. 2024) including native PIEZO-like channels of human cardiac cells (Jakob et al. 2021). Although not necessarily specific for PIEZOs (Kinsella et al. 2024), the effects of this toxin support the idea of PIEZOs having roles in the heart because the toxin has protected against ischemic injury (Wang et al. 2016a, 2024), blood pressure dysregulation (Huo et al. 2021), myofibroblast phenotype (Niu et al. 2022), stretch-induced increases in natriuretic peptides (Zhang et al. 2021), atherosclerosis (Yang et al. 2022), sepsis-induced cardiomyopathy (Zhang et al. 2025), and cardiac arrest induced by mechanical impact on the chest (commotio cordis) (Quinn et al. 2017). The small molecule Dooku1, synthesized based on the structure of the PIEZO1 agonist Yoda1 (Syeda et al. 2015; Evans et al. 2018), can also be useful as an inhibitor of endogenous PIEZO1 channels (Kinsella et al. 2024). Dooku1 reduced valve thickness, calcification, fibrosis, and osteogenic differentiation in two mouse models of calcific aortic valve disease, preserving cardiac function (Zhong et al. 2023). Dooku1 also inhibited laminar flow–induced latent heparanase release from endothelial cells, which might be useful for improving cardiac function in the context of diabetes comorbidity (Lee et al. 2022; Niu et al. 2025). Benzbromarone, a treatment for gout, and PIEZO1 inhibitor (Liang et al. 2024), is suggested to reduce cardiovascular disease risk and mortality (Kang et al. 2021). MicroRNA and short hairpin RNA strategies might also be considered for reducing PIEZO expression (Huang et al. 2016; Lu et al. 2025).

#### PIEZO1 Activation

A common strategy in PIEZO studies is the application of the PIEZO1 agonist Yoda1 (Syeda et al. 2015; Kinsella et al. 2024). However, Yoda1 effects are not necessarily physiological because the PIEZO1 activation may exceed and in other ways not properly mimic effects of physiological forces. Nevertheless, beneficial lymphatic stimulator effects of Yoda1 have been observed in animals, suggesting promise for PIEZO1 agonists as medicines in lymphedema and other lymphatic drainage problems (Choi et al. 2024; Matrongolo et al. 2024). Effects of this type might be relevant to the heart because of PIEZO1’s role in pericardial effusion (Ranade et al. 2014a; Ludlow et al. 2023), and the suggestion that lymphatic stimulators may accelerate the clearance of damage products and unwanted inflammatory mediators after cardiac ischemia (Cooper et al. 2024). In pharmacology, a drug’s dose and administration are crucial; therefore, while PIEZO1 agonists could generate high risks for the heart such as accelerated valve disease (Zhong et al. 2023), inflammation (Blythe et al. 2019), fibrosis (Bartoli et al. 2022b), and arrhythmia (Rolland et al. 2023; Su et al. 2023), low doses of such agonists may be selective for lymphatics (Choi et al. 2019) and have beneficial cardiac effects (Chen et al. 2025b), and so could yet find a place in cardiac therapeutics.

## Conclusions and Future Perspectives

**Figure 1:**
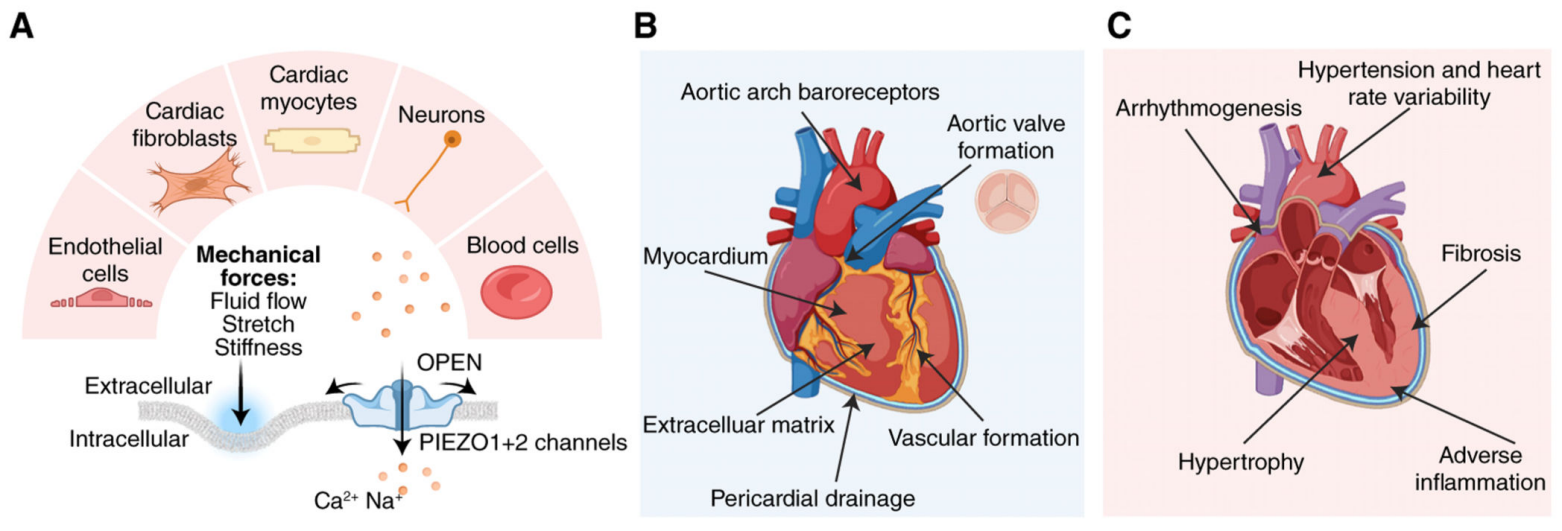
PIEZO force sensors and the heart. (*A, upper*) Cell-type locations of PIEZOs in and around the heart, including but not limited to endothelial cells, cardiac fibroblasts, cardiac myocytes, autonomic and sensory neurons, and blood cells such as red blood cells and monocytes. (*Lower*) Sketches of PIEZO channel indicating the activation by mechanical forces such as shear stress from fluid flow, membrane tension from cell stretch, and viscoelasticity from extracellular matrix stiffness. (*B*) Summary of the suggested physiological roles of PIEZOs in the heart including aortic valve formation, vascular formation, pericardial drainage, aortic baroreceptor pressure sensing and reflex regulation of blood pressure, protection from myopathy, and formation and regulation of extracellular matrix. (*C*) Summary of the suggested implications of altered PIEZO expression in pathophysiology including in reduced reflex regulation of heart rate and blood pressure, increased arrhythmia such as atrial fibrillation, hypertrophy, fibrosis, and adverse inflammation. (Figure created with BioRender.)

We conclude that PIEZO force sensing is pivotal across many aspects of the heart (Fig. 1). It enables the heart to form its valves, vasculature and pericardial drainage. It enables baroreceptor regulation of the heart rate and influences the heart’s size and stiffness. The two PIEZOs interplay and may overlap in their roles. Despite this new knowledge, there is still much we do not understand. Of particular importance is the identification of how and why the activity of the different PIEZOs is appropriately controlled and integrated with associated proteins and lipids, other ion channels, and other molecular signaling of the diverse cell types of the heart across the complex cardiac architecture. In the anticipated future research, there are likely to be important opportunities to be realized from the application of new advanced laboratory techniques in combination with sophisticated computational simulations of PIEZOs in their native force and lipid environments, integrated with the greater complexity of the heart as a whole (Brown et al. 2024). Despite the importance and interest in hearts throughout the animal kingdom, there is, we suggest, a most pressing need for knowledge about PIEZOs specifically in the human heart. Much of what we know about heart PIEZOs derives from animal studies, yet the anatomical structures, rhythms, and mechanics of animal hearts often differ substantially from those of human hearts.

There is relatively little known about PIEZOs in the etiology of natural cardiac pathophysiology, yet the study of this is likely to be important because the induction of cardiac disease–like events in animals has robustly shown adverse changes in PIEZO expression, with strikingly upregulated PIEZO1 in cardiac stress and injury, for example. There are important questions to address about the roles of PIEZOs in physiological stress conditions such as exercise training and pregnancy and in the common unsolved human heart disease problems of old age like cardiac ischemia, cardiac inflammatory disease, heart failure, and cardiac arrhythmia. Recent studies in dogs suggested an adverse role of PIEZO2 in neuronal ganglia of epicardial fat pads, driving atrial fibrillation (Li et al. 2025). PIEZOs could be explored as potential biomarkers of heart disease, for example through the analysis of blood samples and tissue biopsies, and they could be targets in new therapies.

The suggestion of PIEZO-targeted medicines will require careful considerations that recognize the broad expression and roles of PIEZOs in the heart and beyond. There will need to be progress with PIEZO pharmacology, especially for the selective inhibition of PIEZOs and the modulation of PIEZO2 (Kinsella et al. 2024). Sufficient PIEZO selectivity of agents may require targeting modalities other than or along-side small molecules, such as peptides, antibodies, or nucleotide-targeting approaches. It will need to be borne in mind that chronic and systemic administration of PIEZO modulators could come with safety risks due to the wide expression and core functions of PIEZOs in mechanical sensing across the body. Novel tissue-and cell-targeting approaches may be considered in seeking to overcome this latter caveat. Existing non-PIEZO therapies may also be explored for their potential to reduce unwanted PIEZO activity by reducing adverse mechanical strains on the heart.

This is an exciting new research topic that promises much for our fundamental understanding of the heart, and which could lead to new strategies for improving human health.

## Footnotes

7 UniProt gene and protein names are used throughout.
